# How point‐of‐care HbA_1c_ testing changes the behaviour of people with diabetes and clinicians – a qualitative study

**DOI:** 10.1111/dme.14219

**Published:** 2020-01-08

**Authors:** J. A. Hirst, A. J. Farmer, V. Williams

**Affiliations:** ^1^ Nuffield Department of Primary Care Health Science University of Oxford Radcliffe Observatory Quarter Oxford UK; ^2^ National Institute for Health Research (NIHR) Oxford Biomedical Research Centre Oxford UK; ^3^ School of Nursing Nipissing University North Bay ON USA

## Abstract

**Aim:**

To explore adults with diabetes and clinician views of point‐of‐care HbA_1c_ testing.

**Methods:**

Adults with diabetes and HbA_1c_ ≥ 58 mmol/mol (7.5%) receiving HbA_1c_ point‐of‐care testing in primary care were invited to individual interviews. Participants were interviewed twice, once prior to point‐of‐care testing and once after 6 months follow‐up. Clinicians were interviewed once. A thematic framework based on an a priori framework was used to analyse the data.

**Results:**

Fifteen participants (eight women, age range 30–70 years, two Asians, 13 white Europeans) were interviewed. They liked point‐of‐care testing and found the single appointment more convenient than usual care. Receiving the test result at the appointment helped some people understand how some lifestyle behaviours affected their control of diabetes and motivated them to change behaviours. Receiving an immediate test result reduced the anxiety some people experience when waiting for a result. People thought there was little value in using point‐of‐care testing for their annual review. Clinicians liked the point‐of‐care testing but expressed concerns about costs.

**Conclusions:**

This work suggests that several features of point‐of‐care testing may encourage behavioural change. It helped some people to link their HbA_1c_ result to recent lifestyle behaviours, thereby motivating behavioural change and reinforcing healthy lifestyle choices.

## What's new?

- People with diabetes found having HbA_1c_ measured using point‐of‐care testing more convenient to usual care.
- Receiving an instant result helped people understand how lifestyle behaviours affected their diabetes control.
- Clinicians liked using the point‐of‐care tests but were concerned about costs to the clinic.

**What's new?**

- People with diabetes found having HbA_1c_ measured using point‐of‐care testing more convenient than usual care.
- Receiving an instant result helped people understand how lifestyle behaviours affected their diabetes control.
- Clinicians liked using the point‐of‐care tests but were concerned about costs to the clinic.

## Introduction

Diabetes is a growing healthcare burden with implications for healthcare budgets globally 1, and it imposes a major personal burden on the lives of people managing their condition and attending clinic appointments 2, 3, 4. Monitoring diabetes control involves measuring HbA_1c_ and management with glucose‐lowering medications to reduce the risk of developing complications 5. In the UK, this takes place in primary care and often requires people with diabetes to make two visits to the clinic: first to give a venous blood sample, then returning to get the result and any necessary medication changes. Point‐of‐care testing provides an efficient alternative as the test can take place in the doctor's office using a finger‐prick blood sample giving a result within minutes 6, thus only requiring a single visit to the surgery. Whether this then leads to changes in clinical management or changes in behaviour, for example, improved adherence to appointments or medication, is not yet known.

To date, only a single study has explored views of point‐of‐care HbA_1c_ testing in a primary care setting in the UK 7, 8. Nurses and General Practitioners (GPs) interviewed in that study felt that having the result available for discussion with the patient was a strong advantage, but the usefulness of the result may depend on the nurses' responsibilities and their ability to make management changes. However, in that study, the usual care processes were not changed in some cases because clinicians did not act upon results in the consultation, but continued to see their patients twice. In order for people with diabetes to benefit from point‐of‐care testing, results must be acted upon during the point‐of‐care consultation 9. To determine whether point‐of‐care testing could be effectively implemented during a routine primary care visit in the UK, a feasibility study was carried out, in which the point‐of‐care test result was fed back to the patient during the point‐of‐care consultation. Clinicians were asked to make clinical decisions and take action on the test result within the point‐of‐care consultation 6. Briefly, in this mixed methods study, three GP practices recruited 30 people with diabetes and followed them up over 6 months using point‐of‐care HbA_1c_ testing. Participants received two point‐of‐care tests during follow‐up to collect quantitative data. An embedded qualitative design was used to complement, explain and corroborate findings from the quantitative data collection 10, and to explore both participants' and clinicians' views on point‐of‐care HbA_1c_ testing. The aims of this study were to examine participants' expectations, perceptions and experiences of point‐of‐care HbA_1c_ testing.

## Methods

Three GP practices in the Thames Valley region of the UK were recruited to a feasibility study 6 based on their willingness to participate in the research. Of the 30 adults with type 2 diabetes and HbA_1c_ ≥ 58 mmol/mol (7.5%) who received HbA_1c_ point‐of‐care testing as part of the study 6, 15 were invited for individual interviews. This qualitative study was a smaller exploratory study alongside a larger quantitative feasibility study. The aim of the qualitative study was to explore the perspectives of clinicians and people with diabetes using point‐of‐care testing. We used a pragmatic, mixed method approach in addressing our overall research aim to evaluate the feasibility of point‐of‐care testing in type 2 diabetes care. A pre‐specified sample size of 15 people with diabetes was selected based upon socio‐demographic criteria (age, sex, ethnicity and level of education) and duration of diabetes, with the aim of attaining a maximum variation sample with a wide range of patient experiences within the sample 11. Each invited participant was interviewed twice; once before they experienced point‐of‐care testing and again at the end of the study. All interviews were digitally recorded and transcribed verbatim.

Semi‐structured interviews were conducted using open‐ended questions on participants' expectations (interview 1), views and experiences of point‐of‐care testing (interview 2). Interview questions were developed in collaboration with patient representatives, and focused on knowledge and understanding of HbA_1c_, attitudes to testing, appointment length, and understanding and expectations of point‐of‐care testing in the first interview. Questions in the second interviews after point‐of‐care testing covered the process of point‐of‐care appointments, convenience, experience and views of point‐of‐care testing. The second interview included some of the same questions as the first interview so as to pick up possible changes in responses, views or behaviour; questions which were specific to the experience of point‐of‐care testing were also asked. Themes that had arisen during the first interview with a particular participant, which may have concerned care or behaviour, were reintroduced in the second interview to explore those issues further. This enabled a before‐and‐after comparison of each participant's views and experiences regarding point‐of‐care testing. A participant's responses to themes discussed in both interviews are reported together as verbatim quotations in the text to illustrate differences or similarities in their views before and after receiving point‐of‐care testing. The interview questions have been included in the supporting information, in Boxes S1 and S2.

At the end of the study, the clinician from each site who carried out the point‐of‐care consultations was interviewed for their perspective on using point‐of‐care testing and its effect upon the clinical consultation and clinical decision‐making. A thematic framework analysis based on an a priori framework, developed from existing research 12, was used to analyse the interview data; this enabled the use of both predefined and emergent themes to guide analysis 13, 14, and new topics which emerged from the data were incorporated into the framework 15. Although the framework analysis is not aligned with a particular epistemological or theoretical approach, it provides a highly systematic structure with which to analyse qualitative data, and allows comparison across cases and within individuals 13, 16. Participants' interviews were coded once both interviews were complete, and data from both interviews were coded in sequence to retain the linked nature of the interviews for each participant to ensure that changes in behaviour, views or perceptions of point‐of‐care testing between the first and second interviews were picked up. Deductive coding was carried out by the lead researcher (J.A.H.) using QSR International NVivo version 10 software with the main themes in mind, although open, inductive coding was also used to ensure that other aspects of the data were captured for future hypothesis‐generating analyses, and that any other relevant themes were picked up. The three clinician interviews were coded together at the end of the study. For these, all aspects of point‐of‐care testing were analysed using a framework based on the advantages and disadvantages of point‐of‐care testing, and its impact on clinical decision‐making, practicalities and patient flow.

Once coding was complete, codes were grouped into related topics, then topics which fitted into the framework themes (convenience, barriers, behaviour and immediate result) were grouped together under the relevant theme. Data charting was carried out using Microsoft Excel; each theme was labelled as a separate tab in an Excel spreadsheet. Data for each participant were kept together with separate columns for participant identification, code, and then verbatim text retrieved from NVivo codes for the first and second interviews as appropriate. The results are presented to highlight the main analytical findings, and quotations are provided to substantiate the findings for each theme 17, 18. Both the age and sex of the participant, as well as a study identification number, are included with each quotation.

Measures taken to ensure the rigour of data collection included involving patient representatives when devising the interview questions and asking open questions whenever possible to allow the interviewee to express their own views. Rigour in the coding process was ensured by having a random sample of coded transcripts double‐checked by a senior qualitative researcher (V.W.). We ensured rigour throughout the research process, drawing on concepts of transparency, credibility, transferability and reflexivity as indicators of research quality in qualitative research 19, 20. Transparency was maintained by keeping a research journal where all decisions on sampling and analytical processes were recorded. We presented research findings to people with type 2 diabetes to ascertain findings related to their own experiences. We remained reflexive by discussing the identified codes and themes within the research team and critically examining the research decisions made, and how we may have influenced the process.

## Results

### Participants' characteristics

The characteristics of the 15 participants recruited to the interview study are presented in Table 1. Eight participants were women, the age range was 30–70 years, BMI range was 28.2–40.7 kg/m^2^, and the duration of diabetes ranged from 1 year 10 months to 20 years. Two participants were Asian, 13 were Europeans; eight participants were in paid employment and two were insulin users. Interviews lasted an average of 30 minutes each, ranging from 8 to 53 minutes. The three clinician interviews with two nurses and one GP lasted between 14 and 27 minutes.

**Table 1:** Profile of participants

Women/men	8/7
Mean age, years	56.9 (range 30–75)
Mean BMI, kg/m^2^	32.4 (range 28.2–40.7)
Duration of diabetes, years	7.7 (range 1.83–20)
Ethnicity	13 European 2 Asian
Highest level of education	Secondary school – 5 College – 5 University or higher ‐ 5
Number in paid employment	8
Insulin use	2

The final analysis of participant interviews identified four themes, which were advantages and convenience, behaviour and motivation, immediacy of result and visibility, and concerns with point‐of‐care testing. The three clinician interviews were coded together at the end of the study to explore advantages, disadvantages, impact on clinical decision‐making and patient flow. The framework of factors which may influence the adoption of point‐of‐care testing and mechanisms by which it may influence behavioural change is shown in Table 2 along with a description of each theme and supporting quotations. The themes were convenience and time‐saving, motivation to improve behaviour, immediacy and visibility of the result, and concerns with missing an annual test.

**Table 2:** Thematic framework for interviews with participants with diabetes

Theme	Description
Advantages and convenience	Convenience to people with diabetes due to single appointment: ‘I think having everything all in one go is a big advantage. You know, going in for your blood test, then going back to discuss it, you know from a personal point of view it's time‐consuming.’ [P03, 50‐year‐old woman]
Behaviour and motivation	Forced people to confront their diabetes and behaviours: ‘It's better…it's better for the patient because the information's there on the spot. They can't say, “Ah [um] that happened last week or, I've changed since the blood test”…It's there in front of them. There's, you know, rabbit in the headlamps, you've got them…The evidence is there so that's what I quite like about it because the evidence is there; there's no, “Well that was 3 weeks ago, I've changed”—that's what's your blood sugar level is there and then, that's your average, so let's address it.’ [P28, 55‐year‐old man]
Immediacy of result and visibility	An instant result helped people to understand how very recent lifestyle behaviours may have contributed to changes in results: ‘[um] And [er] it's a great benefit to have the results straight away and then you can address all your concerns [er] in that appointment, within the same appointment. And [um] also based on the result you get you may have questions about [um] what did I do wrong; I've eaten too many fruits, and you can ask, “Is that OK, should I change the amount of fruit, or should I change the amount of bread and…”, but if you get a result after several days and then you're having to wait another 1 or 2 weeks for the doctor's appointment, you may be…you can't remember everything you want to ask. So I think it's [um]…yeah it's a really good method of doing things yeah.’ [P24, 30‐year‐old woman]
Concerns with point‐of‐care testing	Concerns about accuracy and not receiving their annual test. ‘And that's the lowest I've ever been since I've had diabetes…Which I was pleased and I thought afterwards, “I wonder if that machine's right because it is a new one”’ [P08, 73‐year‐old man]

### Advantages and convenience

During participant interviews, the convenience and time‐saving element of only having to attend a single appointment with point‐of‐care testing emerged as one of its main advantages, for younger people in particular. One 35‐year‐old woman, who has very young children and works at night, explained that it was easier having to only fit one appointment into her busy life.

‘In this busy world. People don't have much time…People have lots of other things to do. So there is just one appointment…which is perfect.’ [P16, after point‐of‐care testing]

Participants also mentioned that not having to take time out of work to arrange or attend follow‐on appointments made it more convenient.

A 47‐year‐old man who works 12 miles from his GP surgery and has children living at home, viewed time‐saving as a major advantage.

‘The immediacy of the results and that having that conversation all in one go would be a huge time saving.’ [P29, after point‐of‐care testing]

Another man, aged 59 years, has had diabetes for nearly 8 years; although his employers are fine about him taking time out of work for his appointments, he felt that the point‐of‐care test appointments fitted into working life much better.

‘Oh it's fantastic this…you didn't have to make another appointment…No, no time off work, it's there instantly…It's a fantastic idea I think.’ [P17, after point‐of‐care testing]

### Behaviour and motivation

The effect of point‐of‐care testing on motivation to change lifestyle behaviours was a major theme which emerged. For some people this arose from a sense of being watched more closely, which prompted them to confront some of their eating or exercise patterns, or helped to reinforce healthier lifestyle choices and adherence to appointments and medication. This was particularly apparent for younger people who were well‐informed about diabetes, and who appeared to engage with the point‐of‐care test result more than older people. One 55‐year‐old participant suggested that receiving more frequent HbA_1c_ tests was an important factor in reminding her of her status and reinforcing good behaviour.

‘If you said 6 months I like to go perhaps mad or something on the 3 months or 4 months, and then you think, “Ooh slow up down, really watch what you're doing,” because you've got your appointment in 2 months' time.’ [P28]

With usual care, people with diabetes may not be able to see a clear link between their behaviour and the HbA_1c_ test result because of the time between the test and getting the result. Some people could convince themselves that their behaviour had improved in the weeks between giving the blood sample and receiving the results of their HbA_1c_ test. Being confronted with a point‐of‐care test result would mean that this was no longer possible (Table 2). Because diabetes can be invisible and often symptomless for many years, this could give rise to feelings of denial about the condition. For example, one 30‐year‐old woman, who had been living with diabetes for nearly 3 years and had high HbA_1c_ for a number of months, explained that if she avoided going for her tests and getting an official result it made it easy for her to ignore her diabetes.

‘I was kind of deluding myself into thinking I'm on top of it; I'm in control, but I think it was obvious that if I were to go up there, check my blood sugar levels it would have been official that I'm not, so I just wanted to avoid that…I was eating really badly and I knew it was…it was really high and [um] I didn't test it very often because I knew the result and I didn't want to face it.’ [P30, before point‐of‐care testing]

At the end of the study, she explained how the point‐of‐care testing motivated her because it was easier to see how her behaviour had made a difference compared with having to wait for a result in usual care.

‘Yeah really helpful to [um]…to have [um]…have something to show that your behaviour is making a difference for you, helpful for you.’ [P30, after point‐of‐care testing]

The face‐to‐face nature of the point‐of‐care encounter facilitates a discussion of the result, which could provide a more meaningful consultation with participants with diabetes. A 50‐year‐old woman, who often forgot to take her tablets, said that she was encouraged to take her medications after getting her HbA_1c_ result in her point‐of‐care appointment:

‘[Um] I was surprised. Yeah because I don't take the medication very well so I've been good this last week. Since I've had the appointment last week I've started taking my medication again and tried to be good…” [P03]

‘And then I think it's very different having being told over the phone, “Oh yes your result is fine,” or they want to come and discuss it. I think having it there in the, you know, in the surgery there and then is sort of, it's in your face isn't it?’ [P03, after point‐of‐care testing]

In summary, some participants felt that receiving the blood test and result in their appointment allowed them to have a more supportive and meaningful discussion with their GP/ nurse that might encourage them to reconsider and change health behaviours, in contrast to receiving a result in a follow‐on appointment or via a telephone call.

### Immediacy of results and visibility

Overall, the immediacy of the point‐of‐care test result evoked favourable responses. There was a perception that the immediate feedback with point‐of‐care testing could reduce the anxiety that some people experience when waiting for an HbA_1c_ test result. One 49‐year‐old woman had recently experienced some stressful episodes in her life, and she felt that controlling her diabetes was really important to her at that time. Having to wait for her results caused her to worry.

‘I think it would be, make a difference because when you wait for a week or whatever, you don't know what's wrong with you. You can't phone up and you're very anxious and things, and that's what I am sometimes think…I hope my blood's alright.’ [P02, before using point‐of‐care testing]

After having the point‐of‐care test she spoke about feeling more confident with knowing her result and getting immediate advice from the nurse.

‘This is excellent because you'll know where you stand and then you can do something about it if your sugar levels up and down…And that's what I think, it's excellent, because sometimes when you wait for a letter through the post you think…or a doctor's going to ring you [um] and you think there's going to be some bad news. But there you can…[the nurse] can say, “Right I think you ought to go on these tablets to sort your sugar level out,” than waiting for the doctor to tell you.’ [P02, after using point‐of‐care testing]

An immediate result made it easier for people to link the result with their recent behaviour, which could help them understand the impact of exercise and diet on their control of HbA_1c_. One 30‐year‐old woman had been struggling to work out which foods she could eat, and those she should avoid. She found that having an instant result helped her to see how her very recent eating habits affected her HbA_1c_. She also pointed out that getting her point‐of‐care test result meant that there was no denying that the result was definitely hers.

Visibility of the HbA_1c_ result with the point‐of‐care test was deemed to be reassuring and reinforcing; however, some people felt that an inclusion of trends would be valuable. A 47‐year‐old man felt that although he may not remember his actual HbA_1c_ result, it would help him if he could see a graph showing where his HbA_1c_ was relative to his target value.

To summarize, the instant result from point‐of‐care testing may help reduce anxiety in some people with diabetes and help others to understand how their recent eating and exercise patterns could affect their HbA_1c_.

### Concerns with point‐of‐care testing

There was some concern about the accuracy of the analysers, particularly if the result was discordant with previous test results. One 73‐year‐old man questioned the accuracy of the analyser when he got his result because it was different to usual (Table 2).

One 57‐year‐old man who was treated with insulin could not see any advantages to point‐of‐care testing. He usually only goes into the surgery to give a blood sample and then receives his result by telephone without having to return to the surgery.

‘I can't see a big benefit in getting it, you know instantaneously rather than a week later because that's generally what we get back, because it's within a week.’ [P26]

Several people expressed concerns that point‐of‐care testing may mean they did not receive their annual test, during which other blood tests are performed. There was a general view that the annual test should be performed as usual, and point‐of‐care testing should only be used for any in‐between HbA_1c_ monitoring, as expressed by this 55‐year‐old woman:

‘So if this is going to be my annual test, my only blood test of the year…Then there would be a concern that they weren't checking anything else, especially as I get older.’ [P28]

Overall, participants' views did not change substantially between the first and second interviews. One participant who had some uncertainties about instrument accuracy felt more confident after receiving point‐of‐care testing. Quotations demonstrating this and other participant views are presented in Box S3 in the supporting information.

### Clinician interviews

The thematic framework used for coding and analysis of the clinician interviews is shown in Table 3. Overall, clinicians had very positive views of point‐of‐care HbA_1c_ testing and did not think that having an immediate result would change their clinical decisions. One nurse pointed out that it would make it possible to monitor people with diabetes more closely when necessary. Another advantage voiced by one clinician was that it could be used for unscheduled visits or interim monitoring:

**Table 3:** Thematic framework for clinician interviews

Theme	Description
Advantages of point‐of‐care testing and convenience	Opportunity for closer monitoring in those who need it. Or unscheduled visits. ‘But sometimes when you do the annual bloods they're not OK. Everything's OK apart from the HbA_1c_ and then I'm often saying to them, “Make this change and do the HbA_1c_ again in 3 months, ” which is a whole appointment…And then come back and see me, so it would take out an appointment and a lab test; you could just bring them back and do it here; that would be a…that's a huge advantage actually.’ [Nurse 2]
Clinical decision‐making and patient care	‘I don't know from our point of view that it made us do anything particularly differently; we could just access results more instantly.’ [Nurse 1]
Practicalities and patient flow	‘So, they were still here between about 20 and 30 minutes, so it actually wasn't, you know it didn't save time…It saved time overall because they didn't have their 10 minute for their blood beforehand but actually the review time didn't change.’ [Nurse 1]
Disadvantages and barriers to point‐of‐care testing	‘So it's probably broadly similar to the cost of the lab tests, isn't it, I would think? [um] The difference is of course we don't pay for lab tests directly. We're going to have a probably of…[um] pathology budget at some point; we don't actually formally have one yet but I'm sure that's coming. [um] So that would end up coming out of our overhead really…Ten thousand pounds a year and that could be an expensive overhead that we really wouldn't rather not have. So, I think that would be my main concern because obviously general practice is a small business and it could end up just costing us a lot of money and for something we could get done through the lab.’ [General Practitioner 1]

‘Yeah, I mean I can see more how it would be with the sort of a more unscheduled visit. So, if someone goes to see [a nurse] and maybe there have been issues with her insulin or maybe that's she's looking at [er] maybe changing their medication, and they come back and talk to her and maybe she'd do it there and then and see how…what progress they're making 2 or 3 months down the line you know, that sort of thing.’ [GP 1]

One nurse explained that it was better that people did not have to wait for a result, which could cause some people anxiety:

‘…for their point of view it's better because they're not sitting stewing, waiting for their results for a week or so beforehand.’ [Nurse 1]

Nurses observed that there was sufficient time in appointments to run the test, discuss the result and give necessary advice or changes to medication during the appointment (Table 3).

‘I allowed 20 minutes but certainly the follow‐up visits they didn't really take that long, so while the test was doing we had a good chance to have a talk, and it wasn't…it was easily done in that time.’ [Nurse 2]

None of the clinicians thought that using point‐of‐care HbA_1c_ testing would result in a change to patient care.

Interviewer: ‘Do you think they'd changed the [um] patient care in any way?’

Interviewee: ‘Not from our point of view really. Perhaps just offering a different way of approaching care that's all. I don't know from our point of view that it made us do anything particularly differently; we could just access results more instantly.’ [Nurse 1]

The main potential barriers to point‐of‐care HbA_1c_ testing were costs and concerns about people with diabetes missing their annual review.

‘The only…the only downside that I could think is when they come for their annual review where they also need other blood tests, so for which we can't incorporate, so that might be a time when we don't use the machine.’ [Nurse 1]

Currently, GP surgeries do not directly pay for laboratory tests ordered, therefore, adoption of point‐of‐care testing would result in additional costs for the surgery (Table 3).

## Discussion

This work is the first in the UK to explore people with diabetes and clinician perceptions of point‐of‐care HbA_1c_ testing when implemented as intended 9. This qualitative study found that the convenience of the single appointment with point‐of‐care testing is a major advantage, and that it may help some people link their recent behaviour to their test result, thereby reinforcing healthy lifestyle behaviours. The immediacy of the result appeared to reduce the anxiety that some people with diabetes experience when waiting for their test result. Both participants with diabetes and clinicians expressed concerns that point‐of‐care testing may mean that their annual diabetes review, in which multiple blood markers are measured, could be missed. Those who are treated with insulin have a different care pathway, which often involves self‐testing of blood glucose and management in hospital clinics. There may not be as many advantages to point‐of‐care testing for those people with diabetes who are already using point‐of‐care devices to monitor blood glucose and self‐adjust their insulin. Clinicians liked using the analyser and believed that it would not affect the clinical decisions being made, only the timing of those decisions. In general, it was felt that point‐of‐care testing should not be used for the annual review. The cost of implementing and delivering the point‐of‐care tests were seen by clinicians to be the largest barrier.

This qualitative study demonstrates how point‐of‐care HbA_1c_ testing may be used by clinicians when implemented in UK primary care settings. It reveals the possible mechanisms by which point‐of‐care testing may change patient behaviour and lead to improved outcomes. Potential barriers and scenarios in which point‐of‐care testing may not work optimally have been identified. These may be helpful in improving the delivery of point‐of‐care HbA_1c_ testing in future.

A strength of this work is that both participants with diabetes and clinicians were interviewed to capture their perspectives. This revealed that the advantages and concerns between the two groups were broadly similar and supportive of this new technology, although both groups highlighted scenarios where usual care may be preferable. A maximum variation sample was sought to acquire a breadth of perspectives, and this was achieved within the sampling framework. However, the three GP practices in which this study took place may not be representative of GP practices across the UK; also, all but two participants were Europeans. It is therefore recognized that the study participants may not fully represent views of the UK population, which may limit the generalizability of these findings. Furthermore, data collection was based on a pre‐specified sample size and did not continue until data saturation was reached, which may have limited the breadth of views represented in this data. Because we were not aiming to develop theory or explore in depth the lived experience of participants, we chose not to use a specific qualitative methodology, such as grounded theory or phenomenology. Our analysis was informed by framework analysis 21, enabling us to explore data both inductively and deductively, common strategies in pragmatic research designs. We chose not to use respondent validation as a way of ensuring credibility, as this is a much debated technique 22, 23. We did not aim for data saturation as this was an exploratory study, therefore our findings may be context‐specific. However, by choosing a purposeful sample and exploring ‘negative’ cases, we have made some attempts to show the transferability of our findings to other, similar, contexts of care.

This study found that a major barrier to point‐of‐care HbA_1c_ testing was cost. Concerns about accuracy, undermining clinical expertise or equipment maintenance raised in another study 24 did not emerge. Cost is likely to remain a barrier in UK primary care without changes to reimbursement models 25, which may be the way things move in the future with the introduction of personalized medicine and new models of care 26, 27. This may only take place once clinical benefit, cost benefit or societal benefit of these technologies have been demonstrated 28, 29.

The findings of this study have highlighted the importance of regular and frequent monitoring of diabetes control and feedback for some individuals to help maintain motivation and healthy lifestyle behaviours. This may be linked to the asymptomatic nature of diabetes, in which a deterioration in HbA_1c_ is invisible to the individual with diabetes and can be easily ignored without monitoring. Monitoring indicators of health status can both give the patient the sense of ‘being watched’, but may also provide them with a reinforcement of healthy lifestyle behaviour, which may encourage them to continue with it. There have been similar findings with tele‐monitoring of blood glucose and blood pressure in diabetes 30, where people with diabetes considered the ‘policing’ aspect of tele‐monitoring as important in managing their condition. Hypertensive people who self‐monitored their blood pressure similarly recognized the importance of regular monitoring because of the silent nature of hypertension and the severe consequences of high blood pressure 31. Importantly, it is unlikely that monitoring alone is the sole motivator for behavioural change: providing pedometers to participants to increase activity levels found no significant change in activity levels; only when a pedometer was provided in combination with a daily step goal did activity levels increase significantly 32.

Some participants indicated how they found the visibility of the point‐of‐care result helpful and reassuring. Diabetes control has been found to improve when people are given a graphic record of trends in their HbA_1c_ over time 33. Some GPs already offer visual feedback of HbA_1c_ to their patients by allowing them to look at the computer screen showing the result, as well as the trends in their diabetes control. Other research has found that giving people with diabetes a health passport, consisting of a record of diabetes control and feedback from appointments, leads to small improvements in HbA_1c_ 34.

This work suggests that there are several important features of point‐of‐care testing that may encourage behavioural change and adherence to appointments or medication. The reinforcing nature of the point‐of‐care testing was a salient factor, along with the 3‐monthly monitoring received by study participants, which may re‐engage some people with diabetes who have a tendency to slip into non‐adherent behaviours. The immediacy of the result helped people link their recent behaviour over the past few weeks to their HbA_1c_ test result. Therefore, regular and frequent appointments may help to reinforce and motivate some people, as may goal‐setting and the use of visual aids to monitor trends.

## Funding sources

Dr Jennifer Hirst is funded by a National Institute for Health Research (NIHR), Doctoral Fellowship for this research project. This publication presents independent research funded by the National Institute for Health Research (NIHR). The views expressed are those of the author(s) and not necessarily those of the NHS, the NIHR or the Department of Health and Social Care. A.J.F. is a NIHR Senior Investigator. J.A.H. and A.J.F. are funded by the NIHR Biomedical Research Centre, Oxford, UK.

## Competing interests

None

## Ethical approval

The study was approved by the Office for Research Ethics Committees Northern Ireland (Reference 14/NI/1127) in December 2014. Substantial amendments were approved in February and November 2015.

## Supporting information


**Table S1.** Demographics of 30 participants recruited to feasibility study.
**Box S1.** Patient interview questions.
**Box S2.** Interview questions for surgery staff.
**Box S3.** Examples between participant views in first and second interviews.
