# Supplementary material for: How point‐of‐care HbA1c testing changes the behaviour of people with diabetes and clinicians – a qualitative study
Source: Diabet Med. 2020 Jan 8;37(6):1008–15. doi: 10.1111/dme.14219 (PMC7318570; doi:10.1111/dme.14219)
Supplement: Supplementary file 1 — Table S1. Demographics of 30 participants recruited to feasibility study. Box S1. Patient interview questions. Box S2. Interview questions for surgery staff. Box S3. Examples between participant views in first and second interviews. [file DME-37-1008-s001.docx]

Supplementary material

Table S1 - Demographics of 30 participants recruited to feasibility study

| Sex | 14 female, 16 male | |
| --- | --- | --- |
| Age (years), mean (range) | 57.8 (30-79) | |
| Ethnicity | 28 white, 2 Asian | |
| Diabetes duration | 6.7 ± 4.3 years | |
| Smoking status | 17 former smokers  13 never smoked | |
| Highest level of education | 7 University  10 College  13 Secondary school | |
| BMI (kg/m^2^) Mean±SD (range) | 32.2±3.5 (25.1 to 40.7) | |
| Living in the same household, n | Alone  Partner  Other family>18  Children<18 | 3  25  9  5 |

**Interview questions**

***Box S1. Patient interview questions***

| Questions specific to pre-POC interview:   1. Could you tell me about your diabetes and how it affects your health on a daily basis? 2. Typically how often do you get your HbA1c tested and how do you get your result? 3. Is there usually a chance to discuss the result with your doctor or nurse? 4. Do you know what your HbA1c *should* be? 5. Who makes the decision about your HBA1c target and how you go about achieving this? 6. Do you feel able to communicate with your GP/nurse about your diabetes and your treatment? 7. Are you happy with the treatment you receive from you GP? 8. How well do you feel you understand your diabetes? 9. How confident do you feel about managing your diabetes yourself? 10. What do you understand about HbA1c point-of-care testing? 11. How do you feel about your GP/nurse using POC testing for measuring your HbA1c? 12. How do you think this type of testing could improve your diabetes care? 13. Can you think of any difficulties this type of testing may bring? 14. How do you expect POC testing to change the management of your diabetes? |
| --- |
| Questions specific to post-POC interview   1. Overall how do you feel about having your HbA1c measured using POC testing over the past 3 months? 2. Has it had any effect on your understanding of your diabetes? 3. Has it had any effect on the way you manage your diabetes? 4. Did the new testing change the way you communicated with your doctor/nurse? 5. Who made the decision about your HbA1c target and what you should do to achieve it? 6. How many surgery staff did you see at each visit and who did what? 7. Were the appointments too long or too short? 8. Did you find the POC testing more or less painful than your usual HbA1c test? 9. What were the main benefits to this type of testing? 10. What were the main difficulties in using POC testing? 11. Overall was the POC test more or less convenient for you than your usual test? 12. Did having appointments every 3 months change the way you behaved in any way? 13. What would you like to see changed? 14. How can we improve your experience? 15. Have you got any other comments or concerns? |

***Clinician interviews***

Interviews with clinicians will be carried out in the GP surgery as face-to-face interviews where possible, otherwise telephone interviews will be conducted. Interview questions are listed in Box 2. The first interview taking place prior to using POC testing will focus on concerns and expectations of POC testing. The second interview will take place at the end of the study and will focus on experiences of using the POC HbA1c devices, views on the best patient care pathway and the interaction with patient. Interviews are expected to last between 30 and 60 minutes and will be audio recorded.

***Box S2. Interview questions for surgery staff***

| Questions specific to pre-POC interview   1. What are your feelings about using POC HbA1c testing? 2. Where will the device be located? 3. Who will be responsible for upkeep and QC? 4. Have you thought about the sequence of patient appointment (care patheway)? 5. What are your feelings about device accuracy? 6. What are your feelings about costs? 7. Do you think using POC testing may change patient care? 8. Do you think using POC testing may change the relationship with the patient? 9. What are your main concerns about POC testing? 10. What do you see as the main benefits of POC testing? |
| --- |
| Questions specific to post-POC interview   1. Overall how do you feel about using the POC HbA1c device over the past months? 2. Where was the device located in your surgery? 3. Who was responsible for care and QC? 4. Who used the device? 5. Was the device easy to use? 6. What are your feelings about the device accuracy? 7. How many surgery staff were involved in each patient appointment? 8. Typically what was the care path for a patient? 9. Were the treatment times appropriate? 10. Was there always an opportunity to discuss the result with the patient? 11. What impact do you think this type of testing had on patient care? 12. Was the POC testing more or less convenient than the usual HbA1c test? 13. What are your views on the costs to the surgery of using these devices? 14. What were the main benefits to this type of testing? 15. What were the main difficulties in using POC testing? 16. What would you like to see changed? 17. How can we improve your experience? 18. Have you got any other comments or concerns? |

***Box S3. Examples between participant views in first and second interviews***

| One participant, 49, had recently experienced some stressful episodes in her life, and felt that controlling her diabetes was really important to her at that time. Having to wait for her results caused her to worry.  *“I think it would be, make a difference because when you wait for a week or whatever, you don’t know what's wrong with you. You can't phone up and you're very anxious and things, and that’s what I am sometimes think … I hope my blood's alright.”* [P02, before using point-of-care testing]  After having the point-of-care test she spoke about feeling more confident with knowing her result and getting immediate advice from the nurse.  *“This is excellent because you'll know where you stand and then you can do something about it if your sugar levels up and down... And that’s what I think, it's excellent, because sometimes when you wait for a letter through the post you think…or a doctor's going to ring you [um] and you think there's going to be some bad news. But there you can…[the nurse] can say, "Right I think you ought to go on these tablets to sort your sugar level out," than waiting for the doctor to tell you.”* [P02, after using point-of-care testing]  A 56-year old woman who works in a care home where they use over-the-counter fingerprick devices for some of the residents felt that the HbA1c POC analyser would be more accurate than the hand-held devices.  *“Mm. Of that machine is it accurate? I mean the one that they use at work it's only a small machine so it's only giving you…but is it accurate, I don’t know. But it's like any machine. Even if I bought a cheap one it will tell you that you are on high, but it wouldn’t be like the proper machine.”* [P22, before POC testing]  *“I was confident yes. I suppose you know why, because I get…at work it's not proper; they just have these machines and, I mean you know those machines that you can get over the counter? … So, those aren't always accurate but I felt that this one was accurate yeah.”* [P22, after POC testing]  A 57-year old man who had type 2 diabetes but was treated with insulin couldn’t see any advantages at all to POC testing. He usually only goes into the surgery to give a blood sample then gets his result over the phone without having to return to the surgery.  *“I can't see a big benefit in getting it, you know instantaneously rather than a week later because that’s generally what we get back, because it it's within a week.”* [P26, before POC testing]  *“Normally says yes, that’s fine or …. The information that comes back is exactly the same, it’s just swifter. What I’m saying is, it doesn’t change the answer, it’s just the speed of the answer… No all it’s doing is getting the answer quicker. The system is working fine and personally they are happy with it and I’m happy with it… from a personal point of view I can see no advantage.”* [P26, after POC testing] |
| --- |
